# Promising Dietary Supplements with Potential Senotherapeutic Effects: Aqueous Extracts from Enzymatically Hydrolysed Hemp Seed Cake Flour and Hemp Seed Protein Concentrate

**DOI:** 10.3390/antiox14060734

**Published:** 2025-06-15

**Authors:** Anthea Miller, Inga Kwiecień, Marek Bednarski, Małgorzata Zygmunt, Jacek Sapa, Mateusz Sablik, Giorgia Pia Lombardo, Concetta Condurso, Maria Merlino, Magdalena Kotańska

**Affiliations:** 1 Department of Veterinary Sciences, University of Messina, 98168 Messina, Italy; anthea.miller@studenti.unime.it (A.M.); maria.merlino@unime.it (M.M.); 2 Department of Pharmacodynamics, Jagiellonian University Medical College, Medyczna 9, 30-688 Cracow, Poland; marek.bednarski@uj.edu.pl (M.B.); malgorzata.zygmunt@uj.edu.pl (M.Z.); jacek.sapa@uj.edu.pl (J.S.); mati.sablik@gmail.com (M.S.); 3 Department of Medicinal Plant and Mushroom Biotechnology, Jagiellonian University Medical College, Medyczna 9, 30-688 Cracow, Poland; inga.kwiecien@uj.edu.pl; 4 Department of Chemical, Biological, Pharmaceutical, and Environmental Sciences, University of Messina, 98166 Messina, Italy; giorgiapia.lombardo@libero.it

**Keywords:** hemp seed cake flour, hemp seed protein concentrate, antioxidant activity, anti-inflammation activity, senotherapeutic effects, dietary supplement

## Abstract

In the present study, the primary by-products of the hemp-seed oil process—hemp seed cake flour and hemp seed protein concentrate—underwent enzymatic hydrolysis using proteases and carbohydrases, either individually or in combination. The effectiveness of these enzymatic treatments in releasing bioactive compounds was evaluated by assessing the antioxidant and anti-inflammatory properties of the aqueous extracts of both hydrolysed and untreated hemp by-products. The aim was to explore their potential senotherapeutic properties and promote their application as dietary supplements. Secondary metabolites such as flavonoids, phenolic acids, and catechins were analysed using high-performance liquid chromatography. Total phenolic, flavonoid, and protein contents were determined using spectrophotometric methods. Scavenging activity (2,2-Diphenyl-1-picrylhydrazyl scavenging assay (DPPH assay)), antioxidant power (Ferric reducing antioxidant power assay (FRAP assay)), and lipid peroxidation-reducing activity (thiobarbituric acid-reactive substance analysis) were assessed through in vitro assays. Possible anti-inflammatory effects were evaluated by assessing haemolysis inhibition. The impact of extracts on albumin glycation induced by exposure to fructose was also determined. To assess the toxicity of extracts, a zebrafish larvae model was employed. All extracts contained significant amounts of phenolic compounds, flavonoids, and proteins, and they exhibited notable activities in reducing lipid peroxidation and stabilising erythrocyte cell membranes. However, they did not significantly influence protein glycation (the glycation inhibition was only in the range of 15–40%). Our research demonstrates the substantial health-promoting potential, including senescence delay, of aqueous extracts from by-products of the hemp-seed oil process, which are available in large quantities and can serve as valuable supplements to support the health of animals, including humans, rather than being discarded as waste from oil production.

## 1. Introduction

Nowadays, the generation of large amounts of waste in food production is a serious problem that has lasting consequences for the environment in the form of pollution, but it is also unethical in the context of global hunger [1,2,3]. For these reasons, correct food waste management must be a significant objective for companies operating in the food industry, but also for consumers [4].

The annual herbaceous plant known as hemp (*Cannabis sativa* L.) belongs to the Cannabaceae family [5]. While in previous centuries, hemp was mostly used for textiles and the seeds were regarded as a waste [6], now, due to their exceptional nutritional qualities, the seeds are currently used as food [7]. The hemp seeds are nutritionally versatile. It is possible to obtain flours applicable in the bakery sector, oils rich in essential fatty acids, proteins with a high biological value and derivatives for fortification, supplementation and nutraceutical purposes. They are a valuable source of protein, essential fatty acids and minerals. The macronutrient composition of hemp seeds from nearly 50 hemp cultivars cultivated for nutritional purposes varies according to the genotypes and environmental factors: typically, the protein concentration does not exceed 30% while containing a high amount of essential amino acids, the dietary fibre content ranges from 30 to 40%, and the lipid content is roughly 25 to 30% [6]. As its polysaccharide component is virtually entirely made up of dietary fibre, the amount of accessible carbs is often rather low and insignificant. Nonetheless, hemp’s interesting nutritional qualities also include the presence of numerous bioactive molecules, including bioactive peptides, sterols and polyphenols, which have an antioxidant effect [8]. Currently, the industrial hemp sector is expanding rapidly, driven by the growing use of hemp seed oil, particularly in the food and pharmaceutical industries, alongside increasing consumer awareness of its health benefits [9]. However, this rising demand also generates significant waste and by-products, requiring effective valorisation strategies. Notably, these by-products represent a valuable source of bioactive compounds, closely resembling the composition of hemp seeds, except for their reduced fat content [10,11].

Cellular senescence is a condition of stable and generally irreversible proliferative halt characterised by a variety of morphological, structural, and functional alterations, including increased expression and secretion of pro-inflammatory and tissue-remodelling mediators [12]. It is triggered by a variety of events and develops in response to potentially harmful signals such as excessively severe or prolonged inflammation, oxidative stress and hyperglycaemia [13]. This condition is critical in tissue physiology and disease; whether activating a senescence state improves or degrades tissue function and homeostasis is entirely context-dependent [14]. Cell senescence acts as a potent tumour-suppressive mechanism, limiting the proliferation of cells at risk of malignant transformation and supporting the repair of acute tissue damage [15], but it also represents a key driver of ageing and age-related diseases such as fibrosis [16], neurodegeneration [17,18], and type 2 diabetes mellitus [19].

The increase of senescent cells can also disrupt tissue homeostasis and contribute to chronic low-grade inflammation that develops with age (inflammaging) [20]. The senescence-associated secretory phenotype (SASP) consists of a complex mixture of cytokines, chemokines, growth factors, proteases and reactive oxygen species (ROS) that may induce senescence in surrounding cells [21]. Accordingly, treatment with compounds termed “senolytics” (that selectively induce senescent cell removal) or “senomorphics” (that attenuate the tissue-destructive secretory state of certain senescent cells), such as quercetin, apigenin, 4,4′-dimethoxychalcone, kaempferol, rutin, berberine, caffeine, caffeic acid, fisetin, catechin, curcumin etc., etc., has been shown to produce many health benefits [22,23].

Ideally, consuming functional nutritional products containing senomorphic compounds could easily help prevent/delay the development of these unfavourable changes leading to senescence and age-related diseases.

As a part of a broader research project aimed at valorising hemp oil production by-products for their potential reintegration into the food supply, the present study focused on enhancing the antioxidant, anti-inflammatory potential of hemp seed cake flour (HSCF) and hemp seed protein concentrate (HSPC), key by-products of the hemp-seed oil process. A biotechnological approach involving carbohydrase and protease hydrolysis was employed to release carbohydrate- and protein-linked bioactive compounds or generate new ones. The effectiveness of these enzymatic treatments was evaluated by assessing the antioxidant and anti-inflammatory properties of aqueous extracts from both hydrolysed and untreated hemp by-products. The aim was to explore their potential for delaying senescence and promoting their use as dietary supplements.

## 2. Materials and Methods

### 2.1. Reagents, Chemicals, and Materials

Analytical grade reagents and chemicals, HPLC grade solvents, or spectrophotometric grade solvents where necessary, and reference substances with 100% purity were purchased from Merck (Merck KGaA, Darmstadt, Germany). Endo-1,4-β-xylanase from *Neocallimastix patriciarum* fungi was bought from Megazyme (Megazyme Ltd., Wicklow, Ireland), whereas Flavorpro^®^ 795MDP and Depol^®^ 40L were kindly provided by Biocatalysts (Biocatalysts Ltd., Cardiff, UK); Finally, HSCF (*Cannabis sativa* L. cv. Futura 75) and HSPC (*Cannabis sativa* L. cv. Futura 75) were gently supplied by Soc. Coop. Molino Crisafulli a.r.l., located in Caltagirone (CT), Sicily, Italy, with certification of compliance with Italian legislation on THC content [24]. As labelled, their composition was as follows: 26% protein, 5.1% carbohydrates, of which 1.8% sugars, 9.6% fat, 46% dietary fibre, <0.05% sodium chloride, for HSCF; 40% protein, 10.7% carbohydrates, of which 1.8% sugars, 7.4% fat, 24% dietary fibre; <0.05% sodium chloride, for HSPC.

### 2.2. Description of Extract Preparation

Hemp seed cake flour (HSCF) and hemp seed protein concentrate (HSPC) arise from the main by-product of the hemp seed oil extraction process. Specifically, HSCF is produced by grinding hemp seed press cake, while HSPC is obtained by refining HSCF to reduce fibre content, thus increasing protein concentration. Both HSCF and HSPC were derived from the monoecious Futura 75 hemp variety. Hemp seed oil is exclusively produced from monoecious hemp varieties, as they offer a superior seed yield compared to dioecious types. Among these, Futura 75 stands out as the most widely cultivated monoecious variety, particularly in Italy [25]. It is especially favoured in Sicily, where its adaptability to the region’s hot, dry climate ensures stable cultivation and high yields, even under water stress conditions. The two hemp seed by-products (HSCF, HSPC) have been enzymatically treated at the Department of Veterinary Sciences, University of Messina, Italy, using three commercial food-grade enzymes, i.e., endo-1,4-β-xylanase, Flavorpro^®^ 795MDP, and Depol^®^ 40L. Flavorpro^®^ 795MDP was a protease preparation containing endopeptidase, exopeptidase and glutaminase activities. Depol^®^ 40L was a broad-spectrum carbohydrase. The HSCF and HSPC samples were hydrolysed using endo-1,4-β-xylanase alone, as well as Flavorpro^®^ 795MDP alone or in combination with Depol^®^ 40L. These enzymes were selected not only to enhance the antioxidant and anti-inflammatory potential of HSCF and HSPC but also to improve their nutritional value, sensory properties and techno-functionality, contributing to overall quality enhancement. Depol^®^ 40L, a broad-spectrum carbohydrase, was specifically chosen to facilitate the release of carbohydrate-linked bioactive compounds and fragment polysaccharides into simple sugars. The endo-1,4-β-xylanase enzyme was selected for its ability to fragment hemicellulose, thus increasing the soluble to insoluble dietary fibre ratio in HSCF and HSPC, improving by-product nutritional and techno-functional properties. Even this enzyme could promote the release of carbohydrate-bound antioxidant compounds. Finally, the application of Flavorpro^®^ 795MDP was expected to provide (1) the release of protein-linked polyphenols, enhancing their bioavailability and boosting the antioxidant properties of the powders, (2) a potential increase in antioxidant activity through the formation of bioactive peptides, (3) the breakdown of bitter peptides, improving the overall flavour profile, and (4) the deamination of glutamine for enhancing the techno-functional properties of HSCF and HSPC. These expected outcomes are substantiated by the existing literature [26,27,28,29].

The hydrolysis conditions were determined based on the specific characteristics of the enzymes, including their optimal temperature and pH range for activity, which, in turn, determined the duration of the hydrolysis process. The optimised experimental conditions for the hydrolysis processes and the by-products and enzymes used are detailed in Table 1, along with the identifier for each hydrolysed sample.

The HSCF and HSPC samples and their hydrolysed derivatives were aqueously extracted (1:10) at room temperature for 3 h under continuous magnetic stirring; the samples were then centrifuged at 4000 rpm for 10 min at room temperature (Centrifuge: XS R-10M, Remi, Maharashtra, India), and the supernatants were used for the analyses below reported. The pH value of the extracts was measured using a benchtop XS pH 8 standard pH meter (XS Instruments, Carpi (MO), Italy) equipped with a Hamilton Polyte Lab electrode (Hamilton, Bonaduz, Switzerland) and the results are presented in Table 1.

### 2.3. RP-HPLC Analysis of Secondary Metabolite

The obtained extracts were filtered through a membrane filter (MCE, 0.2 µm, Alchem, Toruń, Poland) and then analysed using high-performance liquid chromatography (HPLC). The HPLC system was a Merck–Hitachi model paired with a Purospher RP-18e analytical column (4 × 250 mm, 5 μm; Merck) and a diode array detector (DAD). The samples were analysed using the method previously described by Kwiecień et al. 2018 [30]. In summary, the mobile phase consisted of methanol (component I) and 0.5% acetic acid (component II), with a flow rate of 1 mL/min, gradient elution and total analysis time of 90 min.

Qualification and quantification analysis were based on a comparison with 76 reference substances. Flavonoids (43): apigenin, chrysin, cynaroside, isorhamnetin, hyperoside, luteolin, myricetin, narigenin, populnin, quercetin, quercetin 7-glucoside, quercitrin, rhamnetin, robinin, rutoside, scutellarein, vitexin, wogonoside (Sigma-Aldrich^®^, St Louis, MO, USA); apigenin 5-glucoside, apigenin 7-glucuronide, apigenin 4′-rhamnoside, astragalin, avicularin, baicalin, baicalein, diosmetin, isoquercetin, kaempferol, kaempferol 3-glucorhamninoside, kaempferol 3-rhamnoside, kaempferol 7-rhamnoside, kaempferol 4′-glucoside, miquelianin, narirutin, scutellarin, wogonin (ChromaDex, Irvine, CA, USA); apigetrin, isovitexin, gujaverin, oroxylin A, sculcapflavone II, trifolin, vicenin II (ChemFaces, Wuhan, China). Phenolic acids (26): 3,4-dihydroxyphenylacetic acid, 3-hydroxyphenylacetic acid, caftaric acid, caffeic acid, chlorogenic acid, cryptochlorogenic acid, 2-coumaric, 3-coumaric, 4-coumaric acids, dihydrocaffeic acid, ellagic acid, ferulic acid, 4-O-feruloyl-quinic acid, gallic acid, gentisic acid, hydrocaffeic acid, 4-hydroxybenzoic acid, isochlorogenic acid, isoferulic acid, neochlorogenic acid, protocatechuic acid, rosmarinic acid, salicylic acid, sinapic acid, syringic acid, and vanillic acid (Sigma-Aldrich^®^, St Louis, MO, USA). Phenylpropanoid glycosides (2): verbascoside, isoverbascoside (ChromaDex, Irvine, CA, USA). Catechins (5): catechin, epicatechin, epigallocatechin, epigallocatechin gallate, and epicatechin gallate (ChromaDex, Irvine, CA, USA).

### 2.4. Total Phenolic Content

The total phenolic content of extracts was measured using the Folin-Ciocalteu method as described earlier by Patro et al. 2016 [31] with minor modifications. Briefly, 90 µL of extract was mixed with 10 µL of Folin–Ciocalteu reagent. After incubation for 5 min, 100 µL of 2% Na_2_CO_3_ was added. After 10 min of incubation in the dark, absorbance was measured at λ = 730 nm with a plate reader (ThermoFisher Scientific, Waltham, MA, USA). The concentration of phenolic compounds was calculated using the standard curve for gallic acid (from 1 to 0.001 mg; R = 0.995). Results are expressed as gallic acid equivalents: mg per mL of plant extract.

### 2.5. Total Flavonoid Content

The total flavonoid content of extracts was measured using aluminium chloride [32]. Briefly, 120 µL of extract was mixed with 30 µL of 5% NaNO_2_ solution and incubated for 5 min. Then, 300 µL of 10% AlCl_3_ × H_2_O solution was added, and after 6-min incubation, 200 µL of 1 M NaOH and 350 µL of distilled water were added. The absorbance was read at λ = 510 nm with a plate reader (ThermoFisher Scientific, USA), and the total flavonoid content was calculated using quercetin as a standard (from 0.003 to 0.3 mg; R = 0.993). Results are expressed as quercetin equivalents: mg per mL of plant extract.

### 2.6. Total Protein Content

The protein content in the samples was determined using the Lowry method [33]. Briefly, 100 µL of samples were incubated with 100 µL of 1N NaOH solution and 1000 µL of copper reagent (2% Na_2_CO_3_, 2% potassium sodium tartrate, 1% CuSO_4_) for 15 min at room temperature. Then, 100 µL of Folin–Ciocalteu reagent was added and incubated for 30 min at room temperature. Absorbance was read with a plate reader (ThermoFisher Scientific, USA) at λ = 500 nm. Albumin solution was used to construct the standard curve.

### 2.7. In Vitro Assays

#### 2.7.1. Total Antioxidant Activity by Ferric Reducing Antioxidant Power Assay (FRAP)

The capacity of the extract to reduce the ion Fe^3+^ to Fe^2+^ in an aqueous solution containing the 2,4,6-tri(2-pyridyl)-triazine (TPTZ) ligand was assessed as described earlier by Yoshino and Murakami [34] with minor modification [35]. Briefly, 20 µL of the tested solutions were added to 180 µL of reagent (a solution consisting of 10 parts of 0.3 M acetate buffer pH 3.7, 1 part of 0.01 M TPTZ, and 1 part of 0.02 M FeCl_3_×6H_2_O solution). After 10 min of incubation at room temperature, the absorbance was measured at λ = 593 nm. The results were presented as the amount of reduced iron ions (Fe^2+^). FeSO_4_×7H_2_O solution (concentration range 0.1 to 1 mM) was used to construct the standard curve. Ascorbic acid (Vitamin C) was used as a reference compound.

#### 2.7.2. Ability to Scavenge Free Radicals by 2,2-Diphenyl-1-picrylhydrazyl Scavenging Assay (DPPH Assay)

The free radical scavenging effect of the extracts was assessed by the discolouration of an ethanolic solution of 2,2-diphenyl-1-picrylhydrazyl (DPPH) as described earlier by Sharma and Bhat [36] with minor modification [35]. First, a 200 μM solution of DPPH in ethanol was prepared. Then, 180 μL of DPPH solution was added to 20 μL of the tested extracts, and then the samples were incubated in the dark for 10 min. After this time, the absorbance was determined spectrophotometrically at λ = 517 nm with a plate reader (ThermoFisher Scientific, USA). Ascorbic acid was used as a reference compound, for which a standard curve was prepared in the concentration range of 100–1000 μM. The absorbances of the tested extracts were converted into ascorbic acid activity.

#### 2.7.3. Ability to Reduce Lipid Peroxidation by Thiobarbituric Acid-Reactive Substance (TBARS) Analysis

To assess whether the tested extracts have the ability to inhibit lipid peroxidation, the spectrophotometric method described previously by Buege and Aust [37] with minor modifications was used. First, rat brain tissue was collected, suspended in 0.9% NaCl and homogenised to obtain a 0.1% solution. Hemp extracts were first diluted 5 times in water. Then, 100 μL of extract was added to 1 mL of the brain homogenate, and the mixture was incubated for 5 min at 37 °C. After this time, 50 μL of 0.5 mM FeSO_4_ solution and 2 mM ascorbic acid were added to each sample. Samples were incubated for 30 min at 37 °C. Then, 100 μL of 0.2% butylated hydroxytoluene (BHT) solution and 2 mL of a reagent consisting of 3.75% trichloroacetic acid (TCA), 0.37% thiobarbituric acid (TBA), and 50 mM hydrochloric acid were added. All samples were mixed and placed in a water bath for 15 min. After this time, the samples were cooled and centrifuged at 6000 rpm for 10 min. The absorbance was measured at λ = 535 nm. A standard curve was created for 1,1,3,3-Tetraethoxypropane (TEP—a standard malondialdehyde, 1–5 nM). Carvedilol was used as the reference compound at a concentration of 1 mM, for which 100% activity was assumed, and the activity of the tested compounds was determined as a percentage of the carvedilol activity.

#### 2.7.4. Haemolysis Inhibition Assays

##### Isolation and Preparation of Blood Cells

Red blood cells were prepared as described by Parvin et al. [38], with minor modifications [39]. Fresh whole blood was collected from the rat in a glass tube containing 3.2% sodium citrate. The blood sample was then mixed with an equal volume of Alsever’s solution (2% glucose, 0.8% sodium citrate, 0.5% citric acid, and 0.42% NaCl). The sample was centrifuged at 2500 rpm for 5 min, and the supernatant was removed. The cell pellet was washed with 0.9% NaCl and then centrifuged at 2500 rpm for 5 min. This was repeated 3 times until the supernatant became clear and colourless. The cellular component was reconstituted into a 20% suspension (*v*/*v*) with 100 mM phosphate buffer, pH 7.4.

##### Temperature-Induced Red Blood Cell Lysis Assay

Haemolysis was assessed according to the methodology presented by Anwar et al. [40], with minor modifications. The plant extracts were diluted in H_2_O 5- or 50-fold. Reaction mixtures were prepared by mixing a suspension of erythrocytes, plant extracts, and PBS. The maximal lysis sample (control sample) was prepared analogously, but plant extracts were replaced with H_2_O. The zero sample was prepared without plant extracts and without incubating it at high or low temperatures. Ketoprofen (1 mM) and ASA (2 mM) were used as reference compounds. The reaction mixtures were then gently mixed by inverting the tubes. Two temperatures were used to induce lysis: one set of samples was incubated at 50 °C for 30 min, while the other set, prepared in the same way, was incubated at –20 °C. After incubation, the samples were centrifuged at 3000 rpm for 10 min. The absorbance of the supernatant was measured spectrophotometrically at a wavelength of 535 nm. The percentage of haemolysis was calculated using the following formula:

$$H=\frac{As}{Ac}\times 100$$

where *Ac* is the absorbance of the control, and *As* is the absorbance of the sample.

#### 2.7.5. In Vitro Protein Glycation

##### Preparation of Glycated Bovine Serum Albumin (BSA) Samples

The formation of glycated BSA was determined according to the modified method described by Nicosia et al. [41]. In brief, 1 mL of BSA (30 mg/mL in 100 mM sodium phosphate buffer, pH 7.4, containing 0.02% sodium azide to prevent bacterial growth) was incubated with 1 mL of 1.5 M fructose (in 100 mM sodium phosphate buffer, pH 7.4) and 1 mL of the tested extracts. A control sample was prepared using BSA, fructose, plus water. A blank was prepared using only BSA, buffer, plus water. The reaction mixtures were incubated at 37 °C for fourteen days and were afterwards assayed to determine the presence of advanced glycation. Aminoguanidine was used as a reference compound.

##### Advanced Glycation End Products Formation

The fluorescence intensity of the reaction products was determined using the spectrofluorometric detector POLAR star Omega and a plate reader (BMG Labtech, Ortenberg, Germany), with excitation and emission wavelengths being 340 nm and 440 nm, respectively. The results are presented as fluorescence intensity and expressed as per cent of glycation in the control sample (BSA, fructose plus water)—100% of glycation.

### 2.8. In Vivo Toxicity Assay

The zebrafish embryos were acquired through the natural spawning of adult zebrafish (line AB/TL), which were kept in a continuous recirculating closed-system aquarium with a light/dark cycle of 14/10 h at 28 °C. Breeding was conducted at Jagiellonian University’s Zebrafish Core Facility, Institute of Zoology and Biomedical Research, Department of Evolutionary Immunology, in Kraków. Jagiellonian University’s Zebrafish Core Facility is a licensed breeding and research facility (District Veterinary Inspectorate in Krakow registry and Ministry of Science and Higher Education, record numbers 022 and 0057). All experiments were carried out in accordance with the European Community Council Directive 2010/63/EU for the care and use of laboratory animals dated 22 September 2010 (Chapter 1, Article 1 no.3) and the National Journal of Law act dated 15 January 2015 for the Protection of Animals Used for Scientific or Educational Purposes (Chapter 1, Article 2 no.1). All animals were handled in strict conformity with good animal practice guidelines established by relevant national and/or local animal welfare organizations. According to the European Directive 2010/63/EU and Polish law regulations O.J. of 2015, item 266, all procedures performed in the present study with zebrafish and euthanasia do not require Ethics Committee permissions.

To determine whether tested extracts are toxic to zebrafish larvae, 76 h post-fertilized (hpf) larvae (750 pieces, ten per well) were kept in a 6-well plate containing embryonic medium (E3) and exposed to different concentrations of extracts (10×, 50×, and 100× diluted with E3), whereas untreated larvae served as control. Each extract was incubated in duplicate wells. The mortality rate was determined using a microscope (OPTA-TECH MN16, Warszawa, Poland) after 24 h of exposure [42].

### 2.9. Statistical Analysis

All analysis was carried out in three independent experiments and expressed as mean ± standard deviation (SD). Anova, Turkey’s multiple range test, and multivariate analyses (PCA and clustering analysis) were performed using XLstat software, version 2019.1.2 (Addinsoft, Damremont, Paris, France). Non-parametric Kruskal-Wallis’s test and post hoc Dunn’s multiple comparisons were performed using GraphPad Prism Software (San Diego, CA, USA, version 7). Significance was set at *p* < 0.05.

## 3. Results and Discussion

### 3.1. Active Ingredient Content

Hemp seeds, especially those from industrial varieties, are an important source of oil for the food and cosmetics industries and are also a component of dietary supplements. After oil extraction, the remaining seed cake is rich in protein, fibre, and plant metabolites with valuable properties for human health. There is data on the use of both hemp flour and hemp cake as functional foods in the production of bread [43,44,45], ovine and bovine milk yoghurt [46], or as dietary additives in broiler breeding [47]. The parameters studied most frequently are nutritional values, protein content, minerals, and the compositions of amino acids, fatty acids, and tocopherols. The total content of phenolic compounds is also often studied. Their bioavailability depends on the form and degree of processing of the plant material. Water-soluble metabolites, such as phenols, can be released from polysaccharide complexes, cell walls, or other polymeric structures through enzymatic treatment.

Polyphenols, often known as phenolic chemicals, are among the most significant plant secondary metabolites. Dietary polyphenols are a diverse group of aromatic chemicals that contribute to the organoleptic properties of plant-based foods and beverages. Polyphenols found in foods are classified into two types: nonflavonoids and flavonoids. Non-flavonoids are primarily monocyclic acids with two major sub-classes: phenolic acids and stilbenes. Phenolic acids are classified as benzoic and hydroxycinnamic acids. Flavonoids share a nucleus composed of two phenolic rings and an oxygenated heterocycle. They create a wide variety of molecules that can be classified into several categories, including anthocyanins, flavonols, flavanols (e.g., catechins), flavones, and chalcones. Catechins are found in many plants and dietary plant products [48].

**Figure 1:**
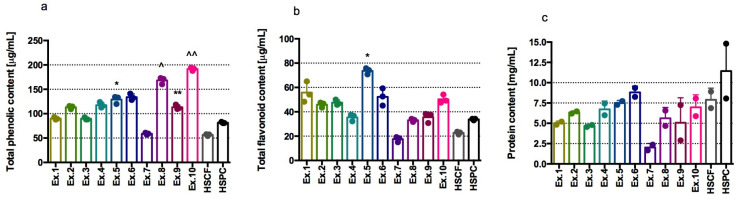
Content of phenolic compounds (**a**), flavonoid compounds (**b**), protein (**c**) in tested extracts Results from three separate experiments in duplicate are presented as mean ± SD, Kruskal-Wallis’s test, Dunn’s post hoc; differences vs. HSCF were considered at * *p* < 0.05, ** *p* < 0.01, differences vs. HSPC were considered at ^ *p* < 0.05, ^^ *p* < 0.01; Ex.1 = HSCF_XL20; Ex.2 = HSPC_XL20; Ex.3 = HSCF_XL30; Ex.4 = HSPC_XL30; Ex.5 = HSCF_XL40; Ex.6 = HSPC_XL40; Ex.7 = HSCF_FP; Ex.8 = HSPC_FP; Ex.9 = HSCF_FP_DL; Ex.10 = HSPC_FP_DL.

In our study, phenolic compounds were found in all extracts; the extracts from both HSPC and its hydrolysed products exhibited higher phenolic content compared to the extracts from HSCF and hydrolysed-HSCF. The results (Figure 1a) indicated that HSPC_FP_DL, HSPC_FP, and HSPC_XL40 were the richest in phenolic compounds, calculated as gallic acid equivalent (the content is from about 0.15 to 0.2 mg/mL). Statistical analysis showed significantly higher contents of these compounds in HSPC_FP and HSPC_FP_DL extracts compared to HSPC extract. Among the HSCF and hydrolysed-HSCF extracts, HSCF_XL40 showed the highest content of phenolic compounds. Statistical analysis showed significantly higher contents of these compounds in HSCF_XL40 and HSCF_FP_DL extracts compared to HSCF extract. Additionally, all extracts from enzymatically treated HSPC and HSCF showed a higher content of phenolic compounds than the extracts from untreated hemp by-products. As expected, the enzymatic treatments involving hemicellulase, protease, and carbohydrase enhanced phenolic content by promoting their release from cellular structures.

In the case of flavonoids, the highest content of these compounds was determined in the HSCF_XL40 extract. In terms of quercetin equivalent, it was slightly below 0.08 mg/mL. Statistical analysis showed a significantly higher content of these compounds in the HSCF_XL40 extract compared to the HSCF extract. The results are presented in Figure 1b. In HSCF_XL20, HSPC_XL40, and HSPC_FP_DL extracts, flavonoids were determined at a level of approximately 0.05 mg/mL quercetin. Unlike phenolic content, the total flavonoid content was higher in the HSCF products after endo-1,4-β-xylanase treatment. This indicates that the release of flavonoids through hemicellulose degradation was more efficient in HSCF than in HSPC. This difference is likely due to the significantly different fibre content of HSPC (24.2%) and HSCF (46.3%). The refining process used to produce the protein concentrate significantly reduces its fibre content compared to the flour. Additionally, the lower pH of endo-1,4-β-xylanase-treated HSCF samples compared to the corresponding HSPC samples may positively influence flavonoid solubility, contributing to the increased levels observed in the hydrolysed HSCF samples.

Nevertheless, the determined level of flavonoids is rather low. This may be related to the chemical nature of the extracts. Flavonoids, especially their aglycones with a small number of hydroxyl groups, exhibit limited solubility in water. The use of a preparation with carbohydrase activity may increase their presence in the extract. As already mentioned, the appropriate processing of the material is of great importance for the bioavailability of metabolites. It should be established experimentally. Based on semi-quantitative HPLC analysis, it was shown that for the Fedora variety, the polyphenol content in hemp flour is 1.5 times higher than in seeds [49]. Other researchers compared four commercial brands of hemp flour and hemp protein concentrate and showed that there were slightly more total phenolics in hemp flour but a higher content of total flavonoids in the protein concentrates. There were also differences between organic and conventionally grown materials [50]. Various enzymatic preparations and methods for extracting active and nutritional compounds were studied in the Finola hemp variety [51]. An important aspect of the analysis was to focus on the content of individual groups of compounds in the solid (sediment) and liquid fractions. Among the polyphenols, proanthocyanidins were studied. Their content was slightly higher in the sediment fraction than in the liquid fraction and depended on the degree of polymerisation of the tannins. Differences in the composition of the fractions, as well as in potential bioavailability, implied different suggested purposes for the obtained products. The enzymatic hydrolysis process also occurs during physiological changes when seeds germinate. It has been shown that during the germination of two varieties (Futura 75 and Secuieni Jubileu), the content of total phenolics increased more than twofold [52].

Hemp seeds are characterised by high protein content, which is a great advantage. According to various authors, the protein content in seeds ranges from 21 to 35.9% of the fresh weight [44,53,54,55,56]. However, hemp cake flour’s protein content is even higher than the seeds from which it is produced [49]. It is also two to three times higher than in wheat flour [44,57].

HSPC and hydrolysed-HSPC extracts had a protein content higher than HSCF and hydrolysed-HSCF. The highest amount of protein was determined in HSPC, HSPC_XL40, HSCF, and HSCF_XL40 extracts. The results are presented in Figure 1c.

Protein content in enzyme-treated products can vary depending on the production process conditions and is difficult to compare. Additionally, the dilution of the tested sample also affects the protein content. In the studies of the Finola variety [51], it was shown that the protein was mainly in the precipitate. The commercially available protein concentrates studied by Absi et al. [43] had 46–47% protein, while the protein isolates in the work of Noelia et al. [58] had 96.46% protein.

The extracts showed the presence of metabolites from the flavonoid, catechin, and phenolic acid groups, which were identified based on the standard compounds available. These were luteolin, kaempferol, apigenin, quercetin, and quercetin glycosides (rutoside and isoquercetin); *p*-hydroxybenzoic, caffeic, ferulic, and *p*-coumaric acid (simple phenolic acids); chlorogenic and neochlorogenic acids (depsides); catechin and epicatechin (Table 2).

The dominant metabolite group in all extracts was catechins. Both catechin and epicatechin were the main metabolites detected. Their content ranged from 294 to 116 µg/mL for catechin and from 86 to 16 µg/mL for epicatechin, respectively. Among the phenolic acids, the depsides were the most abundant, up to 22 µg/mL of extract. Other phenolic acids and flavonoids were determined in much lower, even trace amounts.

Catechins show versatile antioxidant properties that are comparable to or exceed those of flavonoids and phenolic acids, as demonstrated by various analytical methods [48]. Catechins operate through multiple mechanisms. In vitro, catechins scavenge free radicals, chelate metal ions, and inhibit prooxidant enzymes. In vivo, they indirectly stimulate antioxidant enzymes by inducing phase II detoxification enzymes and regulating signalling pathways [59]. Taking into account the content of the metabolites identified by HPLC, HSPC_FP_DL extract was the richest in catechins, whereas HSPC_XL20, HSPC_XL30, and HSPC_XL40 extracts were the richest in phenolic acids; additionally, the extracts from HSCF and HSPC hydrolysed by using 1,4-β-xylanase were the richest in flavonoids. However, the level of estimated flavonoids was very low. As expected, the applied enzymes led to the release of polyphenol compounds linked to both polysaccharides and proteins, significantly enhancing the concentration of these bioactive compounds in all hydrolysed samples compared to the untreated ones.

In the literature, some information regarding the phytochemical profile of phenolic and polyphenolic compounds in hemp seeds can be found. Siano et al. [49] observe that seeds and flour from cv. Fedora exhibits a very similar chromatographic pattern dominated by lignanamides. During the study of seed germination of two varieties (Futura 75 and Secuieni Jubileu), compounds from the phenolic group were identified in the seeds. These included N-trans-caffeoyltyramine, cannabisin A, cannabisin B, and p-hydroxybenzoic acid, with cannabisin A being quantitatively dominant [52]. In contrast, in hemp seeds and seed cake flour from Moldova, lignanamides were predominant, particularly cannabisin F and grossamide. Regarding aromatic acids, benzoic, salicylic, and protocatechuic acids were identified [44]. In the seeds of the Futura 75 variety, using DAD and MS detection, the authors identified 31 hydroxycinnamic acid amides and lignanamides, 4 phenolic acids, 1 lignan, 2 flavones, and 12 cannabinoids. The dominant compounds were N-trans-caffeoyltyramine and cannabisins. Of the flavonoids, only luteolin glucosides were confirmed [60]. Pap et al. utilised enzyme preparations with protease and endopeptidase activity to process hemp seed cake from the Finola variety. Phenolic compounds such as lignanamides and proanthocyanidins were identified in the prepared extracts [51]. The presence of compounds with a phenolic structure in hemp green parts such as flowers, leaves, or sprouts has also been examined. In the inflorescences of 4 hemp cultivars, phenolic acids, lignanamides, phenolic amides, and flavonoids, including catechins, were detected, with cannaflavin A, B, and catechins being dominant [61]. In sprouts of the Sofia hemp variety, chlorogenic, caffeic, and gallic acids, as well as myricetin, were detected. However, the authors also detected other metabolites in the leaves and flowers. Among these, cannaflavin A and isoquercetin were dominant in leaves and flowers, respectively [62].

The results obtained by researchers depend on many factors, such as the method of analysis, available reference compounds, as well as the plant cultivar and the sample preparation and/or processing. In our case, the metabolites were released as a result of the activity of the enzymes employed (endo-1,4-β-xylanase, Flavorpro^®^ 795MDP, and Depol^®^ 40L). It is also important to note that in the natural state, mature seeds were dormant, and metabolites, e.g., phenolics, were mostly bound to other cell components. The availability of metabolites is a key condition for their detection in the analysed material. Therefore, comparing the obtained results can be complicated.

### 3.2. In Vitro Antioxidant Activity of Extracts

Previous research shows that phenolic compounds, flavonoids, and bioactive peptides present in hemp seeds are well recognised for their beneficial properties, including antioxidant [63,64] and anti-inflammatory activities [6]. The results clearly showed that the antioxidant/reductive or anti-inflammatory activity of the tested extracts may result from a rich array of active compounds (phenolic compounds/flavonoids/peptides), which are part of the composition of the extracts.

The antioxidant, reducing, and anti-inflammatory effects are closely linked to senotherapeutic potential, as senescent cells specifically contribute to increased reactive oxygen species (ROS) levels, accelerating chronic inflammation and autoimmunity. ROS are strongly associated with age-related diseases, including cognitive decline and metabolic syndrome. Antioxidants can modulate cellular senescence by scavenging ROS of mitochondrial origin, reducing senescence-associated mitochondrial dysfunction, and disrupting the feedback loop between DNA damage and progressive senescence, thereby mitigating the effects of the senescence-associated secretory phenotype (SASP) [65].

**Figure 2:**
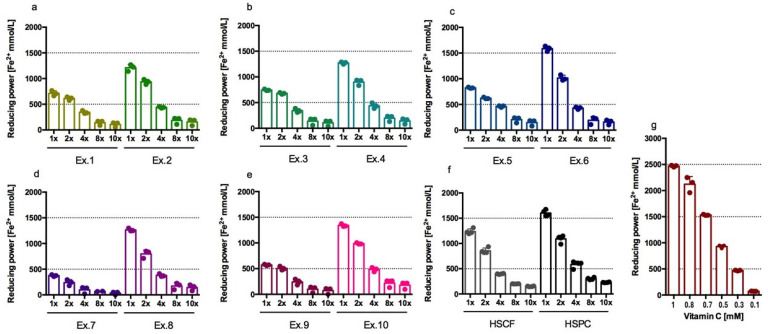
Reductive/antioxidant activity of extracts (FRAP test), Ex.1 = HSCF_XL20, Ex.2 = HSPC_XL20 (**a**); Ex.3 = HSCF_XL30, Ex.4 = HSPC_XL30 (**b**); Ex.5 = HSCF_XL40, Ex.6 = HSPC_XL40 (**c**); Ex.7 = HSCF_FP, Ex.8 = HSPC_FP (**d**); Ex.9 = HSCF_FP_DL, Ex.10 = HSPC_FP_DL (**e**); HSCF, HSPC (**f**); vitamin C (**g**); Results from three separate experiments in duplicate are presented as mean ± SD.

All extracts tested showed a reducing activity that was dependent on dilution. Undiluted HSPC and hydrolysed-HSPC extracts exhibited greater activity than 0.5 mM vitamin C, which reduces approximately 1000 µmol Fe^3+^ to Fe^2+^; on the contrary, undiluted HSCF and hydrolysed-HSCF extracts had a reducing activity lower than 0.5 mM vitamin C. The reducing activity of HSCF extract was about 52% of the activity of 1 mM vitamin C. The results are shown in Figure 2.

Table 3 presents the results of the extract activity as a percentage of Vitamin C activity. The strongest antioxidant activity in this test was demonstrated by HSPC_XL40 and HSPC extracts, whose activity was about 66–69% of the activity of 1 mM vitamin C. The second strongest activity was demonstrated by the HSPC_FP_DL extract, whose activity was about 58% of the activity of 1 mM vitamin C. HSCF_XL40 extract showed the strongest activity among the hydrolysed-HSCF extracts with a value equal to 35% of the activity of 1 mM vitamin C; this value was about 20 percent lower than the activity of HSCF extract.

**Figure 3:**
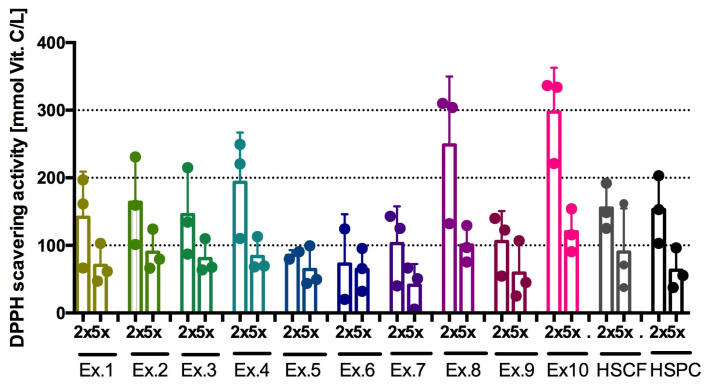
DPPH scavenging/antioxidant activity of extracts (DPPH test). Results from three separate experiments in duplicate are presented as mean ± SD; 2×, 5×—dilution of extract; Ex.1 = HSCF_XL20; Ex.2 = HSPC_XL20; Ex.3 = HSCF_XL30; Ex.4 = HSPC_XL30; Ex.5 = HSCF_XL40; Ex.6 = HSPC_XL40; Ex.7 = HSCF_FP; Ex.8 = HSPC_FP; Ex.9 = HSCF_FP_DL; Ex.10 = HSPC_FP_DL.

The extracts showed weak free 2,2-diphenyl-1-picrylhydrazyl radical (DPPH) scavenging activity. The most active was the HSPC_FP_DL extract, and its activity was comparable to that of vitamin C at a concentration of 300 mM, which is approximately 26% of 1 mM vitamin C activity. The HSPC_XL40 extract demonstrated a more potent effect than the HSPC extract. The results are shown in Figure 3 and Table 4. The HSCF_XL40, HSPC_XL40, HSCF_FP, and HSCF_FP_DL extracts had the weakest effect. The HSCF (2x diluted) and HSPC extracts had comparable activity.

The HSCF_XL40 extract showed the highest antioxidant activity in the lipid peroxidation inhibition test (TBARS levels), amounting to more than 30% of the antioxidant activity of carvedilol. The HSCF_XL20, HSCF_XL30, HSCF_FP and HSCF_FP_DL extracts also showed activity above 20%. The antioxidant activity of both HSPC and hydrolysed-HSCF extracts was less than 20% of that of carvedilol. The results are presented in Table 5. Activity between 25 and 31% was also demonstrated by the reference anti-inflammatory drug—indomethacin, at the used concentrations of 0.1 and 1 mM, respectively. The activity of ketoprofen was two to three times lower and did not exceed 10%. However, acetylsalicylic acid used at a concentration of 0.1 mM inhibited lipid peroxidation with a strength of approximately 65% of the activity of 1 mM carvedilol.

Some previous studies describe the antioxidant activity, including the free radical scavenging effect of extracts from hemp by-products, including hemp protein hydrolysates. The protein isolates from defatted biomass of hemp cake, hemp hulls, and whole hemp seeds exhibit weak yet dose-dependent DPPH radical scavenging properties [44,66,67,68]. Studies have shown that hemp seed protein isolates exhibit a stronger reducing effect, as assessed by the FRAP method, compared to their free radical scavenging ability, measured using the DPPH test [58]. These findings are consistent with our results.

It is important to note that the combination of phenolic compounds can either enhance or diminish the benefits provided by individual bioactive compounds [69]. Phytochemical interactions within extracts may lead to antagonistic effects, which could explain variations in activity across different individual extracts.

Our study indicates that among the HSCF extracts, HSCF_XL40 contained the highest concentration of phenolic acids and a significant level of flavonoids, aligning with its strong ability to inhibit lipid peroxidation. Conversely, HSPC_FP and HSPC_FP_DL extracts possessed the highest phenolic content and exhibited the strongest free radical scavenging capacity (DPPH).

Secondary metabolites in plant cells are produced and usually accumulate in compartments, such as smaller or larger vacuoles. To temporarily attenuate their activity, saccharide moieties are bound through glycosidic linkages. After cell lysis or chemical extraction, they begin to release. Glycosidic linkages are stable in a neutral environment. Acidic or basic pH promotes the hydrolysis of glycosides, releasing the aglycone. The process can be enhanced or conducted enzymatically by glycosidases. Glycosidic bonds are important to the activity and bioavailability of phenolic compounds and polyphenols [70,71,72]. Some phenolic acids, as well as catechins, are incorporated into more complex structures. For example, during cell wall modification, they are located between the polysaccharide fibres of cellulose and hemicellulose. In this case, their release occurs after the application of hemicellulase. The metabolites tested are insensitive to protease activity and are usually not bound to peptides and proteins. This is not a classic mechanism in plant cells. However, proteolysis of the sample results in a mixture of small compounds, amino acids, and others that can potentially interfere with the activity of plant secondary metabolites. They can lead to precipitation and attenuation of phenolic activity.

The antioxidant properties of extracts are determined by their chemical composition, specifically the combination of individual compounds and their redox potential, i.e., the compound’s ability to donate electrons and neutralise oxidation. Antioxidant compounds work through various mechanisms, such as electron transfer, radical scavenging, and metal chelation, and contribute differently to each mechanism. The various antioxidant assays, each with its strengths and limitations [73,74,75], emphasise distinct aspects of antioxidant functionality. Thus, the same extract may exhibit strong activity in one test while showing weaker performance in another, depending on the dominant chemical interactions.

### 3.3. In Vitro Anti-Inflammatory Activity of Extracts

One of the key steps in controlling inflammatory responses is the stabilisation of the lysosomal membrane of activated neutrophils. This leads to a reduction in the release of lysosomal components responsible for inflammation and tissue damage [76]. Extracts/drugs that stabilise the red blood cell membrane can also inhibit the destruction of the lysosomal membrane. Red blood cell membranes and lysosomes have similar components [77]. Stabilisation of red blood cell membranes against hypotonic-induced lysis is considered an indicator of lysosomal membrane stabilisation, which results in anti-inflammatory activity [63]. Red blood cell lysis can also be induced by exposing them to very low (−20 °C) or high (+50 °C) temperatures and studies of the effect on the permeability of red blood cell membranes exposed to these temperatures are often used to screen for the anti-inflammatory activity of plant extracts [38,77,78,79].

**Figure 4:**
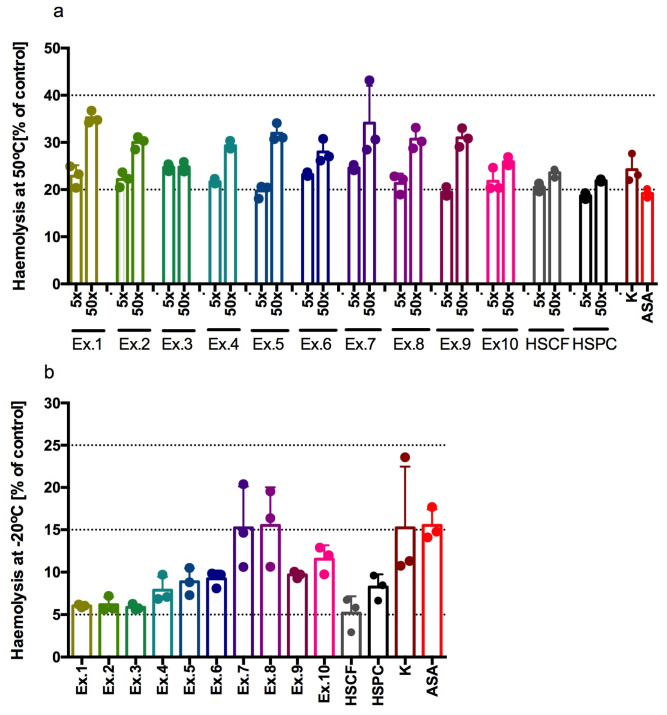
Haemolysis after incubation of extracts with red blood cells in (**a**) high temperature (50 °C), extracts diluted 5× and 50×; and (**b**) low temperature (−20 °C), extracts diluted 50×. Results from three separate experiments in duplicate are presented as mean ± SD; Ex.1 = HSCF_XL20; Ex.2 = HSPC_XL20; Ex.3 = HSCF_XL30; Ex.4 = HSPC_XL30; Ex.5 = HSCF_XL40; Ex.6 = HSPC_XL40; Ex.7 = HSCF_FP; Ex.8 = HSPC_FP; Ex.9 = HSCF_FP_DL; Ex.10 = HSPC_FP_DL, K—ketoprofen 1mM; ASA—aspirin 2mM.

All tested extracts demonstrate activity in inhibiting blood cell haemolysis across two assays. In the haemolysis model induced at 50 °C, samples containing extracts diluted 5x with water showed absorbance levels between approximately 15% and 25% of the control sample, in which the extract was replaced with water (Figure 4a). The HSCF_XL40 and HSCF_FP_DL extracts exhibited activity comparable to HSCF extract and even to 1 mM ketoprofen or 2 mM acetylsalicylic acid. The HSPC extract showed activity comparable to 2 mM acetylsalicylic acid, whereas all hydrolysed-HSCF extracts exhibited slightly less activity. Diluting the extracts 50× with water reduced their erythrocyte cell membrane-stabilising activity, but it remained high. Absorbance readings of the samples ranged from approximately 20% to 35% of the control sample, depending on the extract.

In the haemolysis model induced at −20 °C, samples containing extracts diluted 50x with water showed absorbance at the level between approximately 5% and approximately 15% of the absorbance of the control sample, in which only water was used instead of extracts (Figure 4b). In this assay, all extracts—except HSCF_FP and HSPC_FP—exhibited stronger activity than the reference drugs 1 mM ketoprofen and 2 mM acetylsalicylic acid. However, HSCF_FP and HSPC_FP extracts showed activity comparable to these reference drugs. Our study clearly indicated a high erythrocyte membrane stabilising activity and, therefore, a possible anti-inflammatory effect of all tested extracts.

Cellular ageing induces chronic inflammation and tissue dysfunction, contributing to age-related diseases. In this context, the anti-inflammatory effects associated with the membrane-stabilising properties of the tested extracts are particularly relevant and deserve further investigation to clarify their mechanism of action. These extracts might stabilise erythrocyte membranes by modulating lipid fluidity or influencing protein integration. Certainly, the high levels of phenolic acids in the extracts can protect membrane lipids from oxidation. Reviewing the literature, we did not find any reports on the senolytic and senomorphic effects of aqueous extracts from hemp, including hemp seeds or cakes. However, there are studies describing the senotherapeutic-like properties of other aqueous plant extracts (e.g., green tea, *Solidago virgaurea*, *Lycium barbarum, Zingiber officinale roscoe*, *Desmodium caudatum*) or ethanol plant extracts (e.g., *Isatis tinctoria* L., *Silybum marianum*, *Salvia haenkei*), which, due to the relevant content of active compounds such as phenolic compounds and flavonoids, have an anti-ageing effect [65,80,81,82,83,84]. In general, there is an increasing number of studies on the senotherapeutic-like effects of plant extracts, which have often been traditionally used due to their beneficial properties in this indication. Our screening study shows that aqueous extracts from hemp seed cakes may have senotherapeutic potential due to their antioxidant and anti-inflammatory effects. However, the current study did not directly assess the extracts’ influence on senescence-related pathways. To address this limitation, upcoming research will incorporate enzymatic and cellular assays to gain a more detailed mechanistic understanding of their effects. Furthermore, future investigations will aim to elucidate the bioavailability and metabolic pathways of the active components, providing a more comprehensive insight into their physiological impact. In addition, forthcoming studies will explore optimal formulation strategies to improve stability, efficacy, and potential therapeutic applications.

### 3.4. Effect of the Extracts on Protein Glycation

A primary consequence of hyperglycemia is the formation of advanced-glycation end products (AGEs) and the induction of oxidative damage. AGEs are heterogeneous molecules formed by nonenzymatic modifications of macromolecules such as proteins, lipids, and nucleic acids by glucose, fructose, and pentose. It has been discovered that the number increases with physiological ageing and age-related disorders [85], diabetes mellitus [86], and autoimmune/inflammatory rheumatic diseases [87], including psoriasis [88]. Glycation induces the formation of the β-sheet structure in β-amyloid protein, β-synuclein, etc. Aggregation of the β-sheet structure in the brain creates fibrillar structures, causing Alzheimer’s disease, Parkinson’s disease, amyotrophic lateral sclerosis, familial amyloid polyneuropathy, and others [89]. AGEs can cause oxidative stress, resulting in the generation of proinflammatory cytokines and inflammatory responses [90].

**Figure 5:**
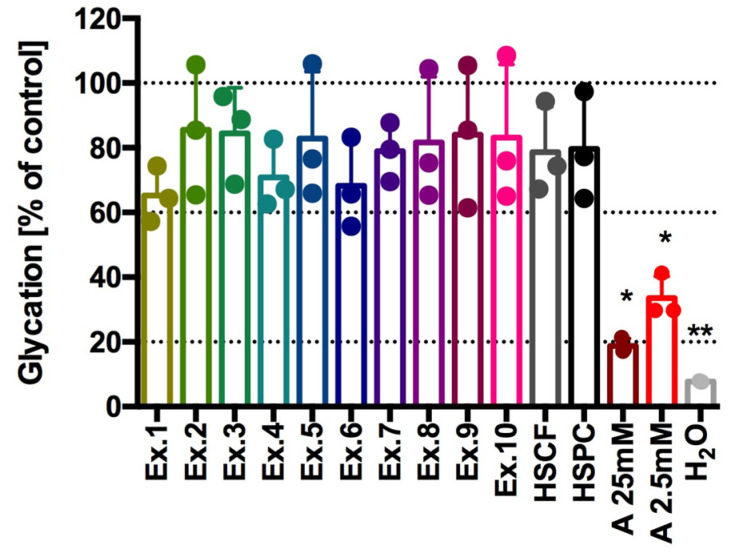
% of glycation after incubation of extracts with BSA+fructose. Results from three separate experiments in duplicate are presented as mean ± SD, Kruskal-Wallis’s test, Dunn’s post hoc; differences vs. control (100% glycation) were considered at * *p* < 0.05, ** *p* < 0.01; 5×—dilution of extract; Ex.1 = HSCF_XL20; Ex.2 = HSPC_XL20; Ex.3 = HSCF_XL30; Ex.4 = HSPC_XL30; Ex.5 = HSCF_XL40; Ex.6 = HSPC_XL40; Ex.7 = HSCF_FP; Ex.8 = HSPC_FP; Ex.9 = HSCF_FP_DL; Ex.10 = HSPC_FP_DL; A—aminoguanidine.

In our study, none of the extracts significantly affected albumin glycation induced by incubation with fructose (*p* = 0.1319). However, it should be noted that, in all tested samples, glycation was approximately 65–85% of the glycation determined in the glycated control sample (BSA + 0.5 M fructose), which means that the extracts were able to inhibit glycation only to a range between 15 and 40%. The aminoguanidine (25 mM and 2.5 mM, the AGEs inhibitor [91]), used as a reference compound, significantly inhibited protein glycation (Figure 5). In the sample incubated with 25 mM aminoguanidine, glycation was at a slightly higher level than the control sample without glycation (BSA + H_2_O), namely 20% vs. 8% of the glycation determined in the glycated control sample (BSA + 0.5 M fructose).

Earlier, a good correlation was shown between the anti-glycating and antioxidant activities of the extracts of ethanol/water extracts of coriander, turmeric, scallion, peppermint, onion, parsley, ginger, curry, scallion, peppermint, onion, and parsley leaves in in vitro studies [92]. Mazzara et al. demonstrated modest efficacy in terms of inhibition of advanced glycation end-product formation by industrial hemp essential oil and its by-products [93]. Undoubtedly, compounds in plant extracts, such as phenolic compounds (including flavonoids) and others, are responsible for their anti-glycation activity. However, this activity is not always evident in various extracts.

Further studies will be carried out to evaluate the extracts’ effects on protein glycation under the influence of other factors, such as glyoxal, methylglyoxal, and glucose. The dicarbonyl compounds like glyoxal and methylglyoxal (two metabolites predominantly produced during glucose metabolism and lipid peroxidation) are precursors of advanced glycation end products, whose presence marks ageing [94].

### 3.5. Comparative Analysis of the Functionality of Extracts

To gain a clearer understanding of the functionality of the various extracts obtained from hemp by-products, i.e., HSCF and HSPC, and their hydrolysates, PCA (Principal Component Analysis) was performed using data on TPC, total flavonoid and protein content, radical scavenging activity (DPPH), ferric reducing antioxidant power (FRAP), lipid peroxidation inhibition (LPI), haemolysis at both 50 °C and −20 °C and protein glycation.

**Figure 6:**
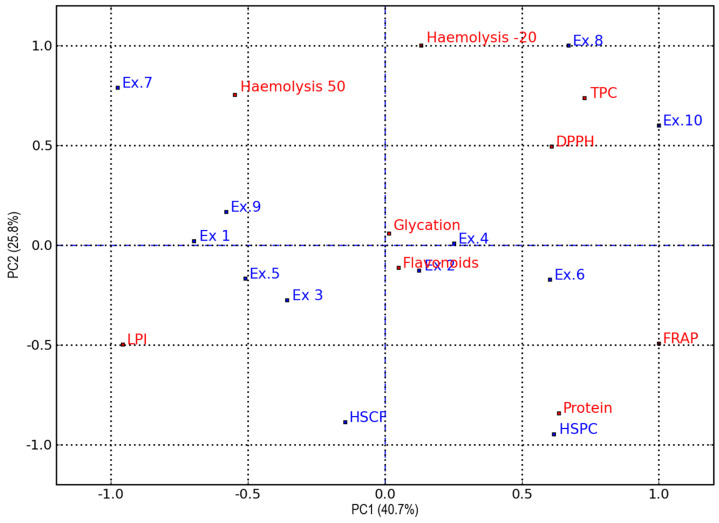
Principal Component Analysis (PCA) score plot (blue spots) and loading plot (red spots) representing the functionality of aqueous extracts from HSCF, HSPC, and their hydrolysates. FRAP = % vit. C activity; DPPH = % vit. C activity; LPI = lipid peroxidation inhibition; Haemolysis 50 = % of haemolysis induced at 50 °C; Haemolysis −20 = % of haemolysis induced at −20 °C; Glycation = % of bovine serum albumin glycation. Ex.1 = HSCF_XL20; Ex.2 = HSPC_XL20; Ex.3 = HSCF_XL30; Ex.4 = HSPC_XL30; Ex.5 = HSCF_XL40; Ex.6 = HSPC_XL40; Ex.7 = HSCF_FP; Ex.8 = HSPC_FP; Ex.9 = HSCF_FP_DL; Ex.10 = HSPC_FP_DL.

Figure 6 displays the PCA score and loading plot for the first two principal components (PCs), which together account for 66.5% of the total variance, with 40.7% explained by PC1 and 25.8% by PC2.

The PCA biplot highlights a clear separation of the samples. Samples were separated along PC1 based on by-product type and along PC2 according to hydrolytic treatment. HSCF and its hydrolysates are positioned on the negative side of PC1 (left side of the graph, i.e., quadrants II and III), while HSPC and its hydrolysates are located on the positive side of PC1 (right side of the graph, i.e., quadrants I and IV). Regarding distribution along PC2, untreated HSCF and HSPC samples are positioned at the bottom of the graph, in quadrants III and IV, respectively, corresponding to the negative side of PC2. Samples treated with endo-1,4-β-xylanase are found near the centre of the graph, yet still on the negative side of PC2 (Ex.5, Ex.3, Ex.2, Ex.6) or at positive values very close to zero (Ex.1, Ex.4). Finally, samples treated with Depol^®^ 40L and/or Flavorpro^®^ 795MDP are positioned on the positive side of PC2. Specifically, Flavorpro-hydrolysed samples (Ex.7, Ex.8) appear in the uppermost part of the graph, while those treated with both Flavorpro^®^ 795MDP and Depol^®^ 40 are located near the endo-1,4-β-xylanase-treated samples in the case of HSCF_FP_DL (Ex.9) and quite far from them in the case of HSPC derivative (Ex.10), which appears on the upper-right near the HSPC_FP sample.

HSPC_FP (Ex.8) and HSPC_FP_DL (Ex.10) samples are separated from the others for their higher TPC and DPPH values. However, they exhibited reduced efficacy in stabilising the erythrocyte cell membrane (positive correlation with haemolysis at −20 °C) and a lower capacity to inhibit lipid peroxidation (negative correlation with LPI). In contrast, extracts obtained from carbohydrase-hydrolysed HSCF (both endo-1,4-xylanase and Depol^®^ 40), as well as untreated HSCF, exhibited low haemolysis at −20 °C and strong lipid peroxidation inhibition, yet showed reduced radical scavenging activity and total phenolic content. The extracts from HSCF, HSPC, and HSPC_XL40 (Ex 6) are associated with elevated protein levels and high FRAP values and negatively correlated to haemolysis at 50 °C. Finally, HSCF_FP demonstrated the lowest functionality, exhibiting the highest haemolysis values and the weakest antioxidant activity, as reflected in its positive correlation with haemolysis at both 50 and 20 °C and its negative correlation with TPC, DPPH, and FRAP values.

**Figure 7:**
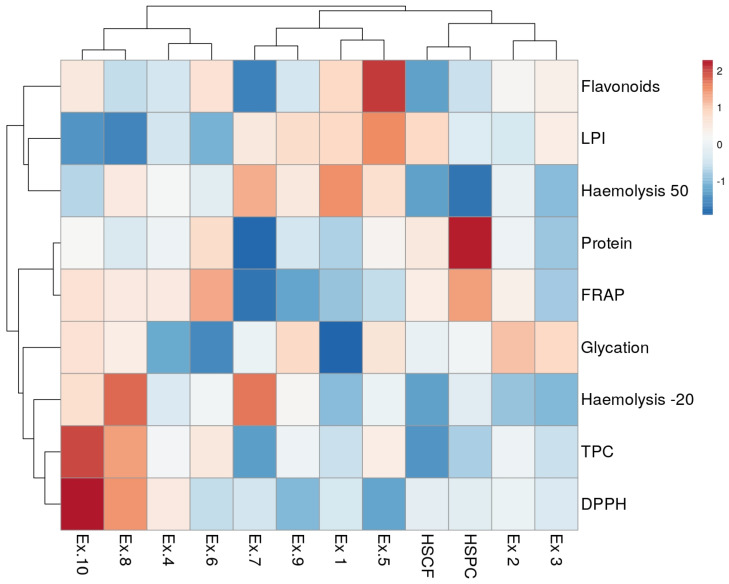
Heatmap visualisation of antioxidant and anti-inflammatory activity among different extracts of untreated and enzymatically treated HSCF and HSPC, and clustering patterns. FRAP = % vit. C activity; DPPH = % vit. C activity; LPI = lipid peroxidation inhibition; Haemolysis 50 = % of haemolysis induced at 50 °C; Haemolysis −20 = % of haemolysis induced at −20 °C; Glycation = % of bovine serum albumin glycation. Ex.1 = HSCF_XL20; Ex.2 = HSPC_XL20; Ex.3 = HSCF_XL30; Ex.4 = HSPC_XL30; Ex.5 = HSCF_XL40; Ex.6 = HSPC_XL40; Ex.7 = HSCF_FP; Ex.8 = HSPC_FP; Ex.9 = HSCF_FP_DL; Ex.10 = HSPC_FP_DL.

Figure 7 presents a heatmap illustrating the relationships between the samples and the various biological or chemical parameters used to assess their antioxidant and anti-inflammatory activity, along with the clustering patterns of both samples and parameters. These highlight groups of samples sharing similar values across measured parameters and groups of parameters exhibiting similar trends across the tested samples.

Sample clustering analysis revealed three distinct groups: (1) all hydrolysed-HSPC samples, except HSPC_XL20 (Ex.2); (2) all hydrolysed-HSCF samples, excluding HSCF_XL30 (Ex.3); and (3) untreated HSCF and HSPC, along with HSPC_XL20 and HSCF_XL30.

Regarding variable clustering, haemolysis 50 and LPI grouped together and then further clustered with flavonoids. Proteins were clustered with FRAP, whereas TPC and DPPH were initially clustered together and then further grouped with haemolysis at −20 °C, glycation percentage and, finally, with the protein-FRAP group.

Hydrolysing HSCF using carbohydrases (endo-1,4-β-xylanase and Depol^®^ 40) enriched the extracts in flavonoids compared to the untreated HSCF (control). These hydrolysed extracts exhibited stronger inhibition of lipid peroxidation, similarly low haemolysis at −20 °C and, limited to Ex.1, a greater glycation inhibition. This suggests that enzymatic hydrolysis enhanced the extract’s ability to mitigate oxidative damage while preserving the anti-inflammatory activity observed in the control sample. In contrast, hydrolysing HSCF with Flavorpro^®^ 795MDP resulted in reduced functionality. The enzyme used, being a mix of proteases, did not target insoluble fibres, such as lignin, hemicellulose, and cellulose, which form the structural components of plant cell walls. Without disrupting cell walls, the action of the proteases was limited, compromising protein hydrolysis. Furthermore, the conditions of the hydrolysis process, such as temperature, time, and water usage, likely contributed to the diminished functionality observed in the Flavorpro-treated sample (Ex.7) compared to the untreated HSCF control.

The effects of enzymatic hydrolysis on HSPC extracts varied depending on the enzyme used. Hydrolysing HSPC using proteases alone (Flavorpro^®^ 795MDP) or in combination with carbohydrases (Depol^®^ 40) resulted in aqueous extracts (Ex.8 and Ex.10) significantly richer in TPC and, in the case of Ex.10, also in total flavonoids compared to the control (untreated HSPC), and with improved radical scavenging activity. However, compared to the control, these extracts showed a reduced ability to inhibit lipid peroxidation, stabilise the erythrocyte membrane and inhibit glycation.

In contrast, hydrolysing HSPC with endo-1,4-β-xylanase increased the flavonoid and phenolic levels, particularly in HSPC_XL40 (Ex.6). Additionally, Radical scavenging capacity (DPPH) was preserved—or even enhanced in HSPC_XL30 (Ex.4)—while ferric reducing antioxidant power (FRAP) remained stable or showed a slight decline. In contrast, lipid peroxidation inhibition decreased. Finally, these extracts exhibited a significant improvement in protein glycation inhibition but demonstrated reduced efficacy in stabilising the erythrocyte membrane at 50 °C while maintaining stability at −20 °C. Thus, in the case of HSPC, which is rich in protein, proteases (Flavorpro^®^ 795MDP) enhanced the antioxidant activity of the hemp by-product, while endo-1,4-β-xylanase helped preserve—or even improve—its anti-inflammatory properties.

Considering the overall results of this study, better outcomes could likely be achieved by using proteases in combination with endoxylanase. This approach would allow for both the enhanced antioxidant properties observed with protease and the improved anti-inflammatory effects induced by endo-xylanase. Furthermore, the combined use of these enzymes suggests a potential synergistic effect, further strengthening both antioxidant and anti-inflammatory activities. Endo-xylanase plays a crucial role in breaking down plant cell walls, thereby facilitating protease access to proteins and enhancing its efficiency.

### 3.6. Toxicity Assay in Zebrafish Larvae

The zebrafish has become a great in vivo model for complementing and expanding the findings of existing studies. Nowadays, it is widely used as an animal model for toxicological and pharmacological screening [95,96], owing to its excellent adaptation to laboratory conditions, its advantageous features [97], and its ability to absorb small molecules through the skin from the surrounding water at all stages of development [98].

**Figure 8:**
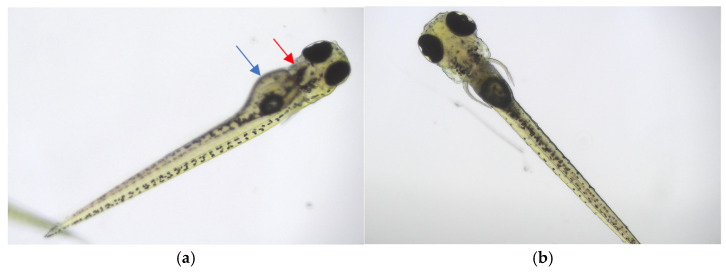
Representative pictures of zebrafish larvae from the toxicity test of the tested extracts, view of the abdominal and lateral part of the larva (**a**), and view of the dorsal part of the larva (**b**). Larvae without visible developmental abnormalities: no yolk oedema and malformation (blue arrow), no cardiac oedema (red arrow), no colour abnormalities, no postural or tail curvatures.

No significant mortality was observed in larvae of Zebrafish treated with the tested extracts (10×, 50×, 100×) over a 24-h period. Only isolated cases of death were noted for some extracts and concentrations, matching the rates observed in control samples. The results are shown in Table 6. All life larvae were without visible developmental abnormalities (no yolk oedema, no cardiac oedema, no colour abnormalities, no postural or tail curvatures). Representative pictures are shown in Figure 8.

These results indicated that the tested extracts were safe even at very high concentrations. Notably, a 10-fold dilution of the extract (the highest used in our studies) significantly alters the buffer, which is suitable for proper larval development. Despite this, our studies showed that survival under exposure to the tested extracts was 80–100%.

This in vivo toxicity test specifically assessed the effects of extracts on zebrafish larval survival. The absence of observable adverse effects suggests the need to broaden toxicity evaluations to include developmental toxicity, malformations, and behavioural impacts. Furthermore, future studies will explore the bioavailability and metabolism of active components to achieve a more comprehensive understanding of their effects.

## 4. Conclusions

This study demonstrated that enzymatic hydrolysis can effectively modulate the antioxidant and anti-inflammatory properties and partial antiglycation activities of hemp seed cake flour (HSCF) and hemp seed protein concentrate (HSPC), key by-products of hemp seed oil production. Given that chronic or excessive oxidative stress, inflammation, and hyperglycemia are well-established drivers of cellular senescence, the bioactivities observed in our extracts suggest their possible modulatory effect on ageing-related processes and potential senotherapeutic properties.

The substrate and specific enzyme used played a crucial role in determining the functional outcomes.

For HSCF, carbohydrases (endo-1,4-β-xylanase and Depol^®^ 40) increased the extracts’ flavonoid content and enhanced their ability to mitigate oxidative damage while preserving anti-inflammatory activity. Conversely, protease treatment alone on HSCF proved less effective, likely due to limitations in cell wall disruption.

For HSPC, protease treatment (Flavorpro^®^ 795MDP) alone or in combination with carbohydrase (Depol^®^ 40) boosted phenolic and flavonoid content and radical scavenging activity but decreased lipid peroxidation inhibition and erythrocyte membrane stabilisation. Interestingly, endo-1,4-β-xylanase treatment of HSPC showed promise in preserving or even enhancing anti-inflammatory properties and significantly improving protein glycation inhibition.

Overall, the findings underscore the potential for valorising hemp oil by-products, transforming waste into functional dietary supplements and promoting sustainability in hemp processing.

These promising findings highlight the value of exploring lesser-used hemp varieties for oil production and/or cultivation under different climatic conditions.

## Figures and Tables

**Table 1:** Optimised process conditions for the hydrolysis of HSCF and HSPC using endo-1,4-β-xylanase, Flavorpro^®^ 795MDP and Depol^®^ 40L.

Sample Identifier ^1^	By-Product Powder	Powder/Water Ratio	Endo-1,4-β-Xylanase	Flavorpro^®^ 795MDP	Depol^®^ 40L	pH	T (°C)	Time (min)	Aqueous Extract pH ^2^
HSCF_XL20	HSCF	01:10	1%w/w fibre	-	-	6.00	50	20	5.67 ± 0.02 e
HSPC_XL20	HSPC	01:10	1%w/w fibre	-	-	6.00	50	20	6.41 ± 0.05 a
HSCF_XL30	HSCF	01:10	1%w/w fibre	-	-	6.00	50	30	5.60 ± 0.06 e
HSPC_XL30	HSPC	01:10	1%w/w fibre	-	-	6.00	50	30	6.50 ± 0.03 a
HSCF_XL40	HSCF	01:10	1%w/w fibre	-	-	6.00	50	40	6.05 ± 0.01 c
HSPC_XL40	HSPC	01:10	1%w/w fibre	-	-	6.00	50	40	6.40 ± 0.02 a
HSCF_FP	HSCF	01:10	-	1%w/w protein	-	6.27	50	180	5.81 ± 0.08 d
HSPC_FP	HSPC	01:10	-	1%w/w protein	-	6.11	50	180	5.96 ± 0.13 cd
HSCF_FP_DL	HSCF	01:10	-	1%w/w protein	2%w/w fibre	5.50	50	240	5.42 ± 0.15 e
HSPC_FP_DL	HSPC	01:10	-	1%w/w protein	2%w/w fibre	5.50	50	240	5.53 ± 0.40 e
HSCF	HSCF	01:10	-	-	-	6.27	50	180	6.27 ± 0.01 b
HSPC	HSPC	01:10	-	-	-	6.11	50	180	6.11 ± 0.01 c

^1^ Sample identifier: abbreviation used to identify the hydrolysate powder samples and their aqueous extracts throughout the manuscript; ^2^ Data are expressed as mean ± standard deviation (n = 3); Different letters within the column indicate statistically significant differences at *p* < 0.05 among pH values of hydrolysed powders by Tukey’s multiple range test. Ex.1 = HSCF_XL20; Ex.2 = HSPC_XL20; Ex.3 = HSCF_XL30; Ex.4 = HSPC_XL30; Ex.5 = HSCF_XL40; Ex.6 = HSPC_XL40; Ex.7 = HSCF_FP; Ex.8 = HSPC_FP; Ex.9 = HSCF_FP_DL; Ex.10 = HSPC_FP_DL.

**Table 2:** Content (µg/mL ± SD) of secondary metabolites in the tested extracts.

HE	NE	C	CG	BA	CA	E	CM	FA	RT	IQ	Q	L	K	A
HSCF_XL20	14.49 ±1.55 a,b,c	203.23 ±16.73 c	10.50 ±2.72 c,d,e	0.10 ±0.03 a,b	6.43 ±0.68 a,b,c	73.17 ±6.74 a,b	3.71 ±0.33 b	1.57 ±0.06 a,b	0.60 ±0.02 g	0.33 ±0.01 g	0.43 ±0.01 d	0.35 ±0.02 d	0.23 ±0.02 d	2.37 ±0.34 a
HSPC_XL20	21.82 ±1.93 d	260.27 ±18.33 a	20.55 ±3.31 f	0.14 ±0.01 b	8.77 ±2.45 b,c	86.93 ±6.21 b	3.97 ±0.30 b,c	2.14 ±0.13 b,c,d,g	0.50 ±0.01 a	0.18 ±0.02 a	0.66 ±0.03 b	0.11 ±0.01 a	0.67 ±0.01 c	7.32 ±0.37 c
HSCF_XL30	16.26 ±1.61 b,c,h	248.63 ±10.20 a,c	12.41 ±3.02 b,d,e	0.11 ±0.01 a,b	6.07 ±0.68 a,b,c,d	53.30 ±4.93 d,e	4.33 ±0.21 b,c	1.89 ±0.26 a,b,c	0.51 ±0.01 a	0.24 ±0.01 b,c,d	0.53 ±0.01 e	0.28 ±0.02 c	6.13 ±0.08 h	2.29 ±0.05 a
HSPC_XL30	19.47 ±2.56 c,d,h	270.50 ±18.60 a	18.11 ±2.00 b,f	0.11 ±0.02 a,b	9.31 ±1.81 b,c	81.61 ±3.24 b	4.98 ±0.89 c	2.44 ±0.30 c,d	0.38 ±0.03 a	0.21 ±0.02 b,c,d	0.68 ±0.03 b	0.11 ±0.02 a	0.47 ±0.04 b	7.19 ±0.15 c
HSCF_XL40	19.93 ±3.04 d,h	294.00 ±24.16 a	13.01 ±2.16 b,e	0.09 ±0.02 a,b	8.15 ±1.17 a,b,c	60.01 ±3.72 a,d,e	1.36 ±0.20 a	0.56 ±0.03 e,f	0.52 ±0.01 f	0.44 ±0.01 e	0.55 ±0.04 e	0.21 ±0.02 b	6.42 ±0.06 i	2.50 ±0.14 a,b
HSPC_XL40	23.68 ±1.67 d	246.63 ±18.91 a,c	18.54 ±2.52 b,f	0.08 ±0.01 a,b	9.83 ±1.51 c	72.99 ±7.24 a,b	1.30 ±0.31 a	1.09 ±0.16 f,g	0.30 ±0.02 a	0.17 ±0.01 a	0.72 ±0.03 b	0.42 ±0.03 e	5.11 ±0.03 g	2.33 ±0.07 a
HSCF_FP	4.30 ±0.75 e	115.88 ±9.98 b	1.25 ±0.25 a	0.19 ±0.01 b,c	1.66 ±0.32 e	15.85 ±3.84 c	1.11 ±0.46 a	2.59 ±0.33 d	0.59 ±0.04 d,e	0.18 ±0.02 a	0.25 ±0.04 a	0.11 ±0.01 a	0.88 ±0.03 e	5.21 ±0.09 d
HSPC_FP	11.44 ±0.70 a,b,g	261.51 ±18.38 a	6.66 ±1.35 a,c,d	0.31 ±0.04 c,d	5.64 ±0.29 a,b,d	45.89 ±2.94 d,f	0.79 ±0.17 a	2.06 ±0.21 a,b,c,d	0.27 ±0.03 g	0.12 ±0.01 f	0.33 ±0.01 c	0.08 ±0.01 a	2.07 ±0.06 f	2.98 ±0.13 b
HSCF_FP_DL	10.21 ±1.35 a,f,g	262.75 ±22.53 a	2.93 ±1.00 a	0.40 ±0.02 d	4.31 ±0.14 a,d,e	37.19 ±3.68 f	0.65 ±0.01 a	1.47 ±0.26 a,g	0.35 ±0.02 c,d	0.25 ±0.02 c,d	0.19 ±0.02 a	0.18 ±0.01 B90	0.37 ±0.01 b	2.61 ±0.09 a,b
HSPC_FP_DL	14.62 ±1.40 a,b,c	426.15 ±25.64 d	4.64 ±0.81 a,c	0.58 ±0.11 e	6.85 ±1.41 a,b,c	62.45 ±5.55 a,e	1.18 ±0.03 a	1.89 ±0.13 a,b,c	0.21 ±0.01 c	0.20 ±0.01 a,b	0.36 ±0.02 c,d	0.10 ±0.01 a	0.72 ±0.01 c	2.68 ±0.03 a,b
HSCF	5.82 ±0.30 e,f	106.34 ±9.92 b	2.12 ±0.77 a	0.01 ±0.00 a	2.22 ±0.28 d,e	9.64 ±1.22 c	0.63 ±0.05 a	0.21 ±0.02 e	0.08 ±0.01 b	0.26 ±0.01 d	0.18 ±0.02 a	0.09 ±0.01 a	0.06 ±0.01 a	2.36 ±0.02 a
HSPC	6.85 ±0.23 e,f,g	124.92 ±8.50 b	3.04 ±1.11 a	0.01 ±0.00 a	4.31 ±1.52 a,d,e	12.84 ±2.04 c	0.73 ±0.11 a	0.58 ±0.01 e,f	0.10 ±0.01 b	0.43 ±0.02 e	0.22 ±0.02 a	0.07 ±0.01 a	0.10 ±0.01 a	2.24 ±0.07 a

Unit: µg/mL of sample; HE—hemp extract, NE—neochlorogenic acid, C—catechin; CG—chlorogenic acid, BA—p-hydroxybenzoic acid, CA—caffeic acid, E—epicatechin, CM—p-coumaric acid, FA—ferulic acid, RT—rutoside, IQ—isoquercetin, Q—quercetin, L—luteolin, K—kaempferol, A—apigenin; Ex.1 = HSCF_XL20; Ex.2 = HSPC_XL20; Ex.3 = HSCF_XL30; Ex.4 = HSPC_XL30; Ex.5 = HSCF_XL40; Ex.6 = HSPC_XL40; Ex.7 = HSCF_FP; Ex.8 = HSPC_FP; Ex.9 = HSCF_FP_DL; Ex.10 = HSPC_FP_DL; letters indicate significant statistical difference (*p* < 0.05).

**Table 3:** Comparison of reductive/antioxidant activity of extracts with Vitamin C activity (FRAP test).

HSCF Extracts	% Vitamin C Activity	HSPC Extracts	% Vitamin C Activity
HSCF_XL20	30.91 ± 2.24	HSPC_XL20	52.56 ± 2.78
HSCF_XL30	32.35 ± 0.67	HSPC_XL30	55.22 ± 0.95
HSCF_XL40	35.77 ± 0.50	HSPC_XL40	68.85 ± 2.23
HSCF_FP	16.29 ± 0.76	HSPC_FP	54.90 ± 1.31
HSCF_FP_DL	25.41 ± 0.58	HSPC_FP_DL	58.26 ± 1.17
HSCF	52.20 ± 2.08	HSPC	66.61 ± 2.44

Results from three separate experiments in duplicate are presented as mean ± SD; Ex.1 = HSCF_XL20; Ex.2 = HSPC_XL20; Ex.3 = HSCF_XL30; Ex.4 = HSPC_XL30; Ex.5 = HSCF_XL40; Ex.6 = HSPC_XL40; Ex.7 = HSCF_FP; Ex.8 = HSPC_FP; Ex.9 = HSCF_FP_DL; Ex.10 = HSPC_FP_DL.

**Table 4:** Comparison of extract scavenging/antioxidant activity to activity of 1 mM Vitamin C (DPPH test).

HSCF Extracts	% Vitamin C Activity	HSPC Extracts	% Vitamin C Activity
HSCF_XL20	11.34 ± 4.78	HSPC_XL20	13.33 ± 4.37
HSCF_XL30	11.62 ± 4.21	HSPC_XL30	16.13 ± 6.37
HSCF_XL40	6.45 ± 1.87	HSPC_XL40	7.77 ± 1.11
HSCF_FP	7.97 ± 6.04	HSPC_FP	21.20 ± 9.30
HSCF_FP_DL	8.31 ± 5.17	HSPC_FP_DL	25.93 ± 6.51
HSCF	19.73 ± 4.27	HSPC	18.96 ± 4.27

Results from three separate experiments in duplicate are presented as mean ± SD. Ex.1 = HSCF_XL20; Ex.2 = HSPC_XL20; Ex.3 = HSCF_XL30; Ex.4 = HSPC_XL30; Ex.5 = HSCF_XL40; Ex.6 = HSPC_XL40; Ex.7 = HSCF_FP; Ex.8 = HSPC_FP; Ex.9 = HSCF_FP_DL; Ex.10 = HSPC_FP_DL.

**Table 5:** Antioxidant activity of the tested extracts and anti-inflammatory drugs: ketoprofen, indomethacin, and acetylsalicylic acid in the lipid peroxidation inhibition test.

Hemp Extracts Diluted 5 Times	% Carvedilol Activity
HSCF_XL20	26.03 ± 0.05
HSPC_XL20	16.54 ± 0.06
HSCF_XL30	22.52 ± 0.06
HSPC_XL30	15.93 ± 0.03
HSCF_XL40	31.89 ± 0.06
HSPC_XL40	11.92 ± 0.04
HSCF_FP	23.41 ± 0.05
HSPC_FP	9.15 ± 0.02
HSCF_FP_DL	25.45 ± 0.03
HSPC_FP_DL	10.20 ± 0.03
HSCF	26.03 ± 0.01
HSPC	17.37 ± 0.03
Carvedilol 1mM	100 ± 0.01
Ketoprofen 1 mM	5.91 ± 0.06
Ketoprofen 0.1 mM	7.18 ± 0.06
Indomethacin 1 mM	30.90 ± 0.02
Indomethacin 0.1 mM	25.51 ± 0.02
ASA 0.1mM	64.76 ± 0.04

Results from two separate experiments in duplicate are presented as mean ± SD; ASA—acetylsalicylic acid Ex.1 = HSCF_XL20; Ex.2 = HSPC_XL20; Ex.3 = HSCF_XL30; Ex.4 = HSPC_XL30; Ex.5 = HSCF_XL40; Ex.6 = HSPC_XL40; Ex.7 = HSCF_FP; Ex.8 = HSPC_FP; Ex.9 = HSCF_FP_DL; Ex.10 = HSPC_FP_DL.

**Table 6:** Zebrafish survivability (expressed as a percentage) in each group.

Sample	Survivability (%)	Survivability (%)	Survivability (%)
Control	90 ± 14.14		
**Hemp Extracts**	**10× Diluted**	**50× Diluted**	**100× Diluted**
HSCF_XL20	100 ± 0.00	100 ± 0.00	100 ± 0.00
HSPC_XL20	100 ± 0.00	100 ± 0.00	100 ± 0.00
HSCF_XL30	85 ± 7.07	90 ± 0.00	90 ± 0.00
HSPC_XL30	100 ± 0.00	100 ± 0.00	100 ± 0.00
HSCF_XL40	100 ± 0.00	100 ± 0.00	100 ± 0.00
HSPC_XL40	100 ± 0.00	100 ± 0.00	100 ± 0.00
HSCF_FP	100 ± 0.00	100 ± 0.00	100 ± 0.00
HSPC_FP	100 ± 0.00	100 ± 0.00	100 ± 0.00
HSCF_FP_DL	100 ± 0.00	95 ± 7.07	100 ± 0.00
HSPC_FP_DL	80 ± 14.14	95 ± 7.07	100 ± 0.00
HSCF	95 ± 7.07	100 ± 0.00	100 ± 0.00
HSPC	85 ± 7.07	100 ± 0.00	100 ± 0.00

Results from two separate wells are presented as mean ± SD; Ex.1 = HSCF_XL20; Ex.2 = HSPC_XL20; Ex.3 = HSCF_XL30; Ex.4 = HSPC_XL30; Ex.5 = HSCF_XL40; Ex.6 = HSPC_XL40; Ex.7 = HSCF_FP; Ex.8 = HSPC_FP; Ex.9 = HSCF_FP_DL; Ex.10 = HSPC_FP_DL.

## Data Availability

The data are included in the manuscript; detailed information is available from the corresponding authors.

## Abbreviations

The following abbreviations are used in this manuscript:

HSCF_XL20	Hemp seed cake flour after 20 min of hydrolysis with endo-1,4-β-xylanase
HSPC_XL20	Hemp seed protein concentrate after 20 min of hydrolysis with endo-1,4-β-xylanase
HSCF_XL30	Hemp seed cake flour after 30 min of hydrolysis with endo-1,4-β-xylanase
HSPC_XL30	Hemp seed protein concentrate after 30 min of hydrolysis with endo-1,4-β-xylanase
HSCF_XL40	Hemp seed cake flour after 40 min of hydrolysis with endo-1,4-β-xylanase
HSPC_XL40	Hemp seed protein concentrate after 40 min of hydrolysis with endo-1,4-β-xylanase
HSCF_FP	Hemp seed cake flour hydrolysed by using Flavorpro^®^ 795MDP
HSPC_FP	Hemp seed protein concentrate hydrolysed by using Flavorpro^®^ 795MDP
HSCF_FP_DL	Hemp seed cake flour hydrolysed by using Flavorpro^®^ 795MDP and Depol^®^ 40L
HSPC_FP_DL	Hemp seed protein concentrate by using Flavorpro^®^ 795MDP and Depol^®^ 40L
HSCF	Untreated hemp seed cake flour
HSPC	Untreated hemp seed protein concentrate
